# Correspondence

**Published:** 1896-03

**Authors:** H. Neher

**Affiliations:** New York


### CORRESPONDENCE.

---

EDITOR JOURNAL OF COMPARATIVE MEDICINE:

For several years I have doubted as to whether the manner of vaccination "in the human," as practised by the human medical practitioner, is proper. It seems to me that vaccination as practised at the present time must surely be behind the times. With the knowledge we have of bacterial life and the mode of infection, it seems to me that vaccination is liable to introduce into the system any and all kinds of bacteria with the virus. Of course, if strict antiseptic measures were followed, there is no danger, but as it is now done, it seems to me to be dangerous. I enclose you an instance which was taken from the daily papers.[1] I will also mention myself.

In the fall of 1873 I was in Richmond, Va.; at the time there was a smallpox scare in the neighborhood where I was staying. The Board of Health ordered every person to be vaccinated. It was done on myself on my left arm. The next day my arm pained me somewhat and kept swelling, paining more and more, swelling more and more; finally it got so bad that a physician was called, and he said it was a case of blood-poisoning, directly due to vaccination. At one time amputation of my arm was seriously considered.

[1] The above case referred to culminated in a law-suit in the Brooklyn courts for heavy damages against the vaccine physician and the Board of Health under whose direction the work was performed.

Three years ago last summer the little daughter of Mr. Charles R., of this city, was vaccinated; being intimate friends of mine, I watched the case. Vaccination was on inside of leg, about midway between knee and groin. Next day leg pained and the child cried, leg swollen, paining more and more. The father asked me to look at it and tell them what I considered the matter. I saw at a glance it was a case of blood-poisoning, and I do not think the child has fully recovered from the effects up to the present time.

I will state one more case. Mr. Michael K., stableman, employed in one of our stables. Vaccinated in 1893 in warm weather. Vaccination on left arm. Next day arm swollen quite a good deal; kept on swelling and paining; had to quit work; was laid up from work seven weeks. Amputation of arm seriously considered. Quite an area of gangrene around the point of vaccination, which sloughed out, leaving a large hole quite down to the bone. This man looks to me to be a case of tuberculosis; never fully recovered.

Now, is it not possible for the point of vaccine virus to become the seat of germ life, and when the individuals are marched up in line, the arm bared and no antiseptic precautions taken, the arm scratched and the virus rubbed into the wound, the sleeve pulled down over the bleeding wound, the patient allowed to go to work, is it not possible, I ask, for germs to be introduced, and is it not dangerous in a district where glanders, tetanus, etc., exists? I am emphatic when I say it is dangerous, and I for one do not approve of the method of vaccination as practised at the present time upon the human subject. Please do not think I am not an advocate of vaccination, I am speaking only about the method. Hoping this will bring about discussion, I am yours, DR. H. NEHER.

NEW YORK, January 20, 1896.

---

The possibilities in the discovery by Professor Röntgen, of the University of Wurzberg, in the ability to detect through the remarkable properties of etheric rays, and thus diagnose tumors, growths, bony enlargements, etc., in the bodies of men and animals, may lead to a perfect revolution in the treatment of many diseased conditions, through the ability thus attained in making an absolute diagnosis.

